# Beyond the Usual Suspects: Unraveling the Etiology of Abdominal Pain with Behcet’s Disease Intestinal Manifestation

**DOI:** 10.5152/tjg.2024.24272

**Published:** 2024-10-01

**Authors:** Idris Kurt, Abdulvahap Kahveci

**Affiliations:** 1 Department of Gastroenerology, Trakya University School of Medicine, Edirne, Türkiye; 2 Department of Rheumatology, Kastamonu Training and Research Hospital, Kastamonu, Türkiye

Dear Editor,

Behcet’s disease (BD), first described and validated by Behçet in 1937, is characterized by vasculitis, which can affect both arteries and veins, with its etiology remaining elusive. This particular ailment predominantly impacts the integumentary and mucosal tissues, eliciting sustained harm across multiple physiological systems. Behcet’s disease manifests as a chronic, relapsing disorder typified by oral and genital aphthae, dermal lesions, and a spectrum of ocular, neurological, and gastrointestinal manifestations. Approximately 3%-16% of BD patients exhibit gastrointestinal tract involvement.^1^ In this instance, we present a case study wherein a patient with BD initially presented with abdominal pain was subsequently diagnosed with intestinal manifestations of the underlying pathology.

A 58-year-old male patient with BD presented with complaints of abdominal pain and loss of appetite for 1 week. Abdominal pain was persistent, highly expressed in the right lower quadrant, and did not change with oral intake or defecation. Upon anamnesis, the patient did not mention any diarrhea, melena, or hematochezia. In his past medical history, he was diagnosed with BD 3 years ago with signs of recurrent oral aphthous ulcers, papulopustular lesions, a positive pathergy test, and a deep vein thrombus in the left lower extremity. Since his diagnosis, he has received azathioprine (2 × 50 mg per day), colchicine (2 × 0.5 mg per day), and acetylsalicylic acid (100 mg per day). On physical examination, there were 3 major ulcers in the oral mucosa, as well as pain in the right lower quadrant of the abdomen. A deep genital ulcer measuring 3 × 4 cm was also observed on the scrotum’s skin (Figure 1). Laboratory tests revealed an increase in CRP (c-reactive protein) and sedimentation rate, 358 mg/dL (range: 0-5) and 94 mm/h (range: 0-20), respectively. The hemogram showed elevated white blood cell count (28.5 × 10^3^), a hemoglobin level of 12.2 g/dL, and a platelet count of 132 × 10^3^. In biochemical tests, liver and kidney function tests were normal. In the abdominal ultrasonography performed for right lower quadrant pain of the abdomen, the walls of the terminal ileum and caecum appeared significantly edematous, and no signs of appendicitis were observed. Abdominopelvic computed tomography revealed intestinal wall thickening at the terminal ileum and cecum levels, as well as mesentery inflammation. Colonoscopy screening revealed a single oval-shaped deep ulcer measuring 3 × 4 cm in size, destructing the ileocecal valve, and sparsely swollen smaller ulcers in the ascending colon (Figure 2). A biopsy was taken from these lesions. The biopsy specimens indicated the presence of vasculitis, characterized by lymphocytic infiltration primarily affecting the walls of veins, along with nonspecific observations. These included an ulcer with regenerative alterations in the villi, active colitis observed in the descending colon, mild crypt distortion, focal crypts, and a slight elevation in lymphoplasmacytic inflammatory cells within the lamina propria. The pathological examination did not detect cytomegalovirus (CMV) or tuberculosis bacilli. The interferon gamma release test, the CMV-DNA test, and the anti-neutrophil cytoplasmic antibody test were also negative. Pathology and rheumatology conferred a diagnosis of intestinal BD. Azathioprine was discontinued. We ordered 1 mg/kg/day of prednisolone and 40 mg/2 weeks of adalimumab for the patients. We continued to administer Colchicine three times per day and applied topical betamethasone 2 × 1 for the scrotal ulcer. At the follow-up visit 1 month later, the patient’s complaint of abdominal pain and genital ulcer had resolved. In laboratory examinations, acute-phase reactants and hemograms were within normal limits. Informed consent was obtained from the patient.

Intestinal Behcet’s disease commonly presents itself within a timeframe of 4-6 years subsequent to the initial diagnosis. Patients frequently exhibit a spectrum of symptoms, including abdominal pain, abdominal distension, diarrhea, and hematochezia. A hallmark characteristic of this condition is the identification of a singular deep ulcer, typically localized within the terminal ileum or ileocecal region, often assuming an oval configuration. This ulceration has the propensity to involve the muscular layer of the intestine, thereby predisposing patients to the potential complication of intestinal perforation.^2^

Other conditions leading to intestinal ulcers must be meticulously excluded, with particular emphasis on tuberculosis (TB) and Crohn’s disease (CD). The exclusion of TB is of paramount importance due to the potential exacerbation of intestinal TB by the immunosuppressive therapy administered for BD. Confirming the diagnosis and assessing the activity of CD involves a comprehensive evaluation that includes clinical, endoscopic, histological, radiological, and/or biochemical markers. Endoscopic manifestations of intestinal TB often encompass annular ulcers and scarred areas with discoloration. Diagnostic modalities include tissue culture, tissue TB PCR (polymerase chain reaction), and interferon-gamma release assays (IGRA), alongside general examinations such as the tuberculin test and chest x-ray. Distinguishing BD from Crohn’s disease can pose challenges as both conditions exhibit extraintestinal manifestations like oral ulcers and arthralgia, common in BD. Endoscopic observations in Crohn’s disease typically entail longitudinal ulcers and a cobblestone appearance, with anal lesions frequently observed.^1,3^ Furthermore, distinctive pathological features such as granulomas, which are observed in tuberculosis and Crohn’s disease, can play a pivotal role in the process of differential diagnosis. Notably, in tuberculosis, granulomas tend to exhibit specific characteristics, notably their size (often exceeding 200 μm), confluence, density (typically exceeding 5-10 high-power fields), and central caseation.^4^

Immunosuppressive agents constitute a cornerstone in the therapeutic management of intestinal BD. Glucocorticoids serve as the primary agents for inducing remission in cases of severe active disease. Following the commencement of glucocorticosteroid therapy, disease-modifying agents are typically incorporated into the treatment regimen. In instances of severe and refractory cases, the consideration of anti-tumor necrosis factor (TNF) antibodies is warranted.^5^

In conclusion, it is imperative for clinicians to maintain a high index of suspicion for intestinal involvement in patients with underlying BD who present with abdominal pain. Prompt and accurate diagnosis is crucial to mitigate potential complications, including perforation, massive bleeding, and unwarranted abdominal surgeries. Adopting a multidisciplinary approach that encompasses the expertise of gastroenterologists, rheumatologists, and surgeons is paramount in ensuring optimal patient care and management.

## Figures and Tables

**Figure 1.**
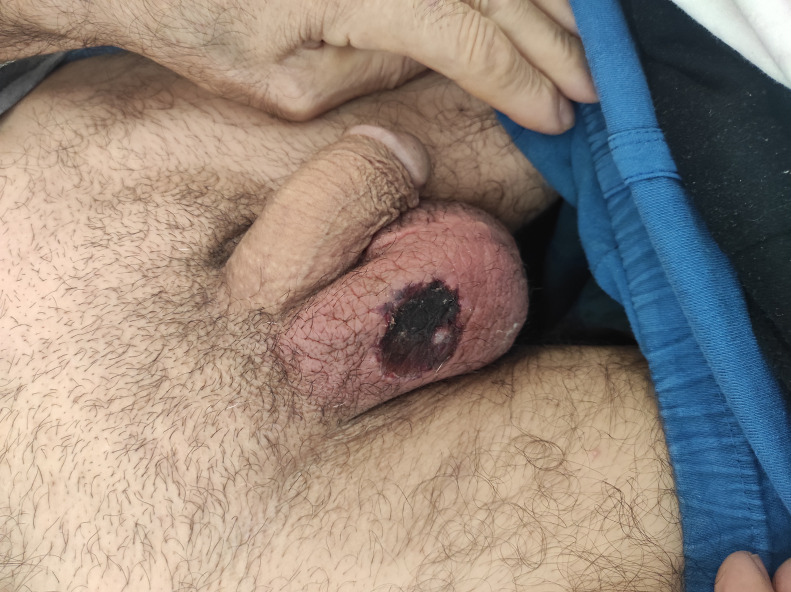
An ulcerative genital lesion with a crust on the skin of the scrotum.

**Figure 2.**
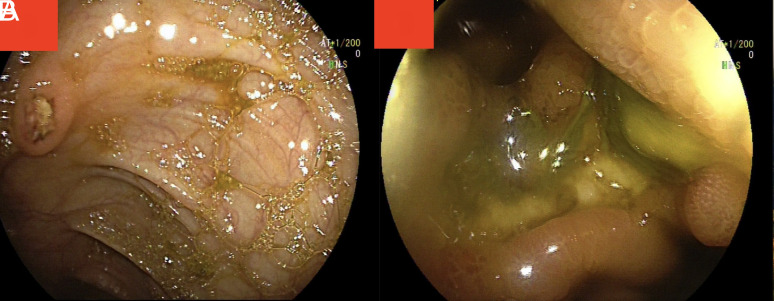
Endoscopic view: sparsely swollen smaller ulcers surrounded by normal mucosa in the ascending colon (A); an oval-shaped deep ulcer measuring 3 × 4 cm in size, destructing the ileocecal valve (B).
